# KCNQ channels in nociceptive cold-sensing trigeminal ganglion neurons as therapeutic targets for treating orofacial cold hyperalgesia

**DOI:** 10.1186/s12990-015-0048-8

**Published:** 2015-07-31

**Authors:** Alaa A Abd-Elsayed, Ryo Ikeda, Zhanfeng Jia, Jennifer Ling, Xiaozhuo Zuo, Min Li, Jianguo G Gu

**Affiliations:** Department of Anesthesiology and Perioperative Medicine, College of Medicine, University of Alabama at Birmingham, 901 19th Street South, BMR II 210, Birmingham, AL 35294 USA; Department of Anesthesiology and the Graduate Program in Neuroscience, The University of Cincinnati College of Medicine, PO Box 670531, 231 Albert Sabin Way, Cincinnati, OH 45267-0531 USA; Department of Orthopedic Surgery, Jikei University School of Medicine, 3-25-8 Nishi-Shinbashi, Minato-ku, Tokyo, 105-8461 Japan; Department of Neuroscience and High Throughput Biology Center, Johns Hopkins University School of Medicine, 733 N. Broadway 311 BRB, Baltimore, MD 21205 USA; Department of Anesthesiology, University of Wisconsin School of Medicine and Public Health, Madison, WI USA; GlaxoSmithKline, New York, NY USA; Department of Pharmacology, Hebei Medical University, 361 East Zhongshan Road, Shijiazhuang, 050017 Hebei China

## Abstract

**Background:**

Hyperexcitability of nociceptive afferent fibers is an underlying mechanism of neuropathic pain and ion channels involved in neuronal excitability are potentially therapeutic targets. KCNQ channels, a subfamily of voltage-gated K^+^ channels mediating M-currents, play a key role in neuronal excitability. It is unknown whether KCNQ channels are involved in the excitability of nociceptive cold-sensing trigeminal afferent fibers and if so, whether they are therapeutic targets for orofacial cold hyperalgesia, an intractable trigeminal neuropathic pain.

**Methods:**

Patch-clamp recording technique was used to study M-currents and neuronal excitability of cold-sensing trigeminal ganglion neurons. Orofacial operant behavioral assessment was performed in animals with trigeminal neuropathic pain induced by oxaliplatin or by infraorbital nerve chronic constrictive injury.

**Results:**

We showed that KCNQ channels were expressed on and mediated M-currents in rat nociceptive cold-sensing trigeminal ganglion (TG) neurons. The channels were involved in setting both resting membrane potentials and rheobase for firing action potentials in these cold-sensing TG neurons. Inhibition of KCNQ channels by linopirdine significantly decreased resting membrane potentials and the rheobase of these TG neurons. Linopirdine directly induced orofacial cold hyperalgesia when the KCNQ inhibitor was subcutaneously injected into rat orofacial regions. On the other hand, retigabine, a KCNQ channel potentiator, suppressed the excitability of nociceptive cold-sensing TG neurons. We further determined whether KCNQ channel could be a therapeutic target for orofacial cold hyperalgesia. Orofacial cold hyperalgesia was induced in rats either by the administration of oxaliplatin or by infraorbital nerve chronic constrictive injury. Using the orofacial operant test, we showed that retigabine dose-dependently alleviated orofacial cold hyperalgesia in both animal models.

**Conclusion:**

Taken together, these findings indicate that KCNQ channel plays a significant role in controlling cold sensitivity and is a therapeutic target for alleviating trigeminal neuropathic pain that manifests orofacial cold hyperalgesia.

## Background

Many clinical conditions including dental procedures, traumatic injury, tumors, and chemotherapy can result in chronic trigeminal nerve injury and degeneration [1]. These often lead to the development of trigeminal neuropathic pain that manifests as cold allodynia/hyperalgesia and mechanical allodynia in orofacial regions [2, 3]. Cold allodynia/hyperalgesia is a severe pain state triggered by innocuous or mild noxious cold temperatures. The trigeminal neuropathic pain constitutes a huge health problem because of its severity, special location, and resistance to conventional treatment [4]. Therefore, there is an imperative need to identify therapeutic targets for effectively treating this intractable trigeminal neuropathic pain.

Recent studies have demonstrated that cold stimuli are mainly transduced by TRPM8, an ion channel that is expressed in both trigeminal ganglion (TG) and dorsal root ganglion (DRG) neurons [5, 6]. TRPM8 can be activated by cooling temperatures below 28°C and also by menthol, an active ingredient of peppermint [5, 6]. Previous studies by us and others have shown that TRPM8 channels are expressed on both non-nociceptive and nociceptive cold-sensing neurons, suggesting that TRPM8 channels are involved in sensing both innocuous and noxious cold under physiological conditions [7, 8]. Several electrophysiological differences have been identified between nociceptive and non-nociceptive cold-sensing neurons. For example, nociceptive cold-sensing neurons express TTX-resistant voltage-gated Na^+^ channels; action potentials of these neurons are slow and each has a hump in its repolarization phase [9, 10]. On the other hand, non-nociceptive cold-sensing neurons only express TTX-sensitive voltage-gated Na^+^ channels and they fire fast action potentials without humps [9]. In both nociceptive and non-nociceptive cold-sensing neurons, TRPM8 starts to be activated at cooling temperatures below 28°C [5, 6, 11]. Therefore, there must be mechanisms to prevent nociceptive cold-sensing neurons from being excited by innocuous or overexcited by mild noxious cold temperatures under physiological conditions.

KCNQ channels, a subfamily of voltage-gated K^+^ channels activated at low voltages near resting membrane potentials (−50 to −60 mV) [12], may play a role in controlling the excitability of nociceptive cold-sensing neurons if KCNQ channels are expressed on these sensory neurons. Five KCNQ subunits including KCNQ1 to KCNQ5 have been identified and 4 of them (KCNQ2-5) are found to be expressed in the CNS and PNS neurons [12]. DRG neurons are shown to express KCNQ2, KCNQ3, and KCNQ5, but functional KCNQ channels are thought to be mainly KCNQ2/3 heteromeric channels and they are believed to underlie the M-type outward K^+^ currents (M-currents) recorded in DRG neurons [13–15]. Because they are activated near resting membrane potentials, KCNQ channels would counteract membrane depolarization and serve as a brake to limit membrane depolarization and control neuronal excitability. Although this idea has been tested in many CNS neurons and also in some PNS neurons [12], little attention has been paid to KCNQ channel’s potential role in controlling the excitability and cold sensitivity of nociceptive cold-sensing neurons.

The expression and function of KCNQ channels have not been extensively studied in trigeminal ganglion neurons although M-currents were recorded from TG neurons [16]. In DRG neurons, previous studies have shown that KCNQ channel subunits KCNQ2 and KCNQ3 are expressed in nociceptive DRG neurons and their expression is down-regulated after nerve injury and in bone cancer in animals [13–15, 17]. These studies have further shown that the KCNQ channel down-regulation is associated with the increase of excitability in nociceptive DRG neurons [15, 17]. Unfortunately nociceptive cold-sensing neurons were not examined in these studies and it is currently unknown if these nociceptive neurons express KCNQ channels or not. Although TG neurons have many functional similarities to DRG neurons, TG neurons have properties that distinguish them from DRG neurons. For example, 15% of TG neurons were found to be TRPM8-expressing cold-sensing neurons, twice the percentage of TRPM8-expressing cold-sensing neurons in DRGs [5]. Thus, the functions of KCNQ channels in cold-sensing TG neurons should be studied directly.

One important advance in research on KCNQ channels has been the identification of small molecule compounds that can potentiate KCNQ channels [18]. There are a series of compounds that can selectively enhance KCNQ channel functions, including retigabine, flupirtine, NH6, meclofenamic acid, etc. [18]. Retigabine has been shown to be effective in suppressing seizure activity in human patients and was recently approved for clinical use [19]. Retigabine has also been found to potentiate KCNQ channels in DRG neurons and relieve carrageenan-induced inflammatory pain in animals [13]. However, it is currently unknown whether KCNQ channel potentiators can effectively treat cold allodynia/hyperalgesia.

In the present study, we examined the expression of functional KCNQ channels in nociceptive cold-sensing TG neurons and determined the roles of these channels in controlling the excitability of TG neurons. We also evaluated the effect of a KCNQ channel potentiator on orofacial cold allodynia/hyperalgesia in animals with trigeminal neuropathic pain produced by oxaliplatin or infraorbital nerve chronic constrictive injury (ION-CCI).

## Methods

### Trigeminal ganglion neuron preparations

Male adult Sprague–Dawley rats (250–350 g) were used. Animal care and use conformed to National Institutes of Health guidelines for care and use of experimental animals. Experimental protocols were approved by the University of Cincinnati Institutional Animal Care and Use Committee. Dissociated trigeminal ganglion (TG) neurons were prepared in a manner similar to the preparation of dissociated DRG neurons as described previously [20]. In brief, rats were deeply anesthetized with isoflurane (Henry Schein, NY, USA) and sacrificed by decapitation. TGs were rapidly dissected out bilaterally in Leibovitz-15 medium (Mediatech Inc. VA, USA) and incubated for 1 h at 37°C in minimum essential medium for suspension culture (S-MEM) (Invitrogen, Grand Island, NY, USA) with 0.2% collagenase and 0.5% dispase and then triturated to dissociate neurons. The dissociated TG neurons were then plated on glass coverslips pre-coated with poly-d-lysine (PDL, 12.5 µg/ml in distilled H_2_O) and laminin (20 µg/ml in Hank’s Buffered Salt Solution HBSS, BD bio-science), and maintained in MEM culture medium (Invitrogen) that also contained nerve growth factor (2.5 S NGF; 10 ng/ml; Roche Molecular Biochemicals, Indianapolis, IN, USA), 5% heat-inactivated horse serum (JRH Biosciences, Lenexa, KS), uridine/5-fluoro-2′-deoxyuridine (10 µM), 8 mg/ml glucose, and 1% vitamin solution (Invitrogen). The dissociated cells were maintained in an incubator at 37°C with a humidified atmosphere of 95% air and 5% CO_2_. Cells were used between 3 and 5 days after the dissociation.

### Patch-clamp recordings from cold-sensing trigeminal ganglion neurons

Coverslips with TG neurons were placed in a 0.5-ml microchamber, mounted on an Olympus IX70 inverted microscope (Olympus, USA), and continuously perfused with a normal bath solution at 2 ml/min. The normal bath contained (in mM) 145 NaCl, 5 KCl, 2 MgCl_2_, 2 CaCl_2_, 10 glucose, 10 HEPES, pH 7.3 and osmolarity of 320 mOsm. Unless otherwise indicated, bath solution was maintained at room temperature of 24°C. Cells were first tested with 100 μM menthol to pre-identify menthol/cold-sensitive cells by using the Ca^2+^ imaging method which we have previously described [21].

For conventional whole-cell recordings, the patch-clamp electrode internal solution contained (in mM) 135K-Gluconate, 5 KCl, 2.4 MgCl2, 0.5 CaCl2, 5 EGTA, 10.0 Hepes, 5.0 Na2ATP, 0.33 GTP-Tris salt, pH was adjusted to 7.35 with KOH and osmolarity was adjusted with sucrose to 320 mOsm. Recording electrode resistance was 3–6 MΩ, and membrane access resistance in the whole-cell configuration was ~10 MΩ and was not compensated. Junction potential between bath and electrode solution was calculated to be 17 mV and was corrected for in the data analysis. Voltage-clamp recordings were performed with cells held at −60 mV. Signals were recorded with an Axopatch 200B amplifier, filtered at 2 kHz and sampled at 5 kHz using pCLAMP 9.0 (Axon Instruments).

### Immunohistochemistry

Adult male rats were anesthetized with ketamine/xylazine (100 mg/kg:10 mg/kg, i.p.), transcardially exsanguinated with heparinized saline, and perfused with 4% paraformaldehyde (PFA) in phosphate buffered saline (PBS). TGs were removed and placed in 30% sucrose in PBS for overnight cryoprotection. The TGs were then embedded in OCT^®^ compound (Baxter Scientific) and 10 μm sections were cut on a cryostat (Leica Biosystems, Buffalo Grove, IL, USA). Sections were thaw-mounted onto slides and allowed to air-dry. They were then encircled with hydrophobic resin (PAP Pen—The Binding Site). The slide-mounted sections were incubated at room temperature for 30 min in a 4% PFA solution and further incubated for 3 h in a mixture solution of 0.4% Triton X-100 and 4% PFA. After 3 rinses with PBS, the sections were incubated 2 h at room temperature with a 2% Triton X-100 solution. The slides were rinsed two times with a 1% goat serum PBS and then incubated for 1 h in a solution of 1:30 normal goat serum in PBS with 0.4% Triton X-100 to block non-specific antibody binding. The sections were incubated with a polyclonal rabbit anti-KCNQ2 antibody (1:400; Alomone Labs, Jerusalem, Israel) over night at 4°C. Following three rinses with 1% goat serum PBS solution, the sections were further incubated with a secondary antibody for 3 h at room temperature. The secondary antibody (1:100 in 1% goat serum PBS solution) was a goat anti-rabbit IgG conjugated with Alexa-488 (Molecular Probes, Eugene, OR, USA). The sections were rinsed three times with 1% goat serum PBS solution, cover-slipped with a glycerol-based anti-photobleach medium. Slices were viewed under an inverted fluorescent microscope (IX-70, Olympus, Tokyo, Japan).

### Orofacial operant behavioral assessment and animal models

Behavioral assessments were conducted on male Sprague–Dawley rats (300–450 g) as described in our previous studies [22, 23]. In brief, animals initially underwent 4–6 sessions of adaptation trainings in 2 weeks using the Ugo Basile Orofacial Stimulation Test System^®^ (Comerio VA, Italy). For each training session, animals were first fasted for a 12-h period. Each rat was then placed in a cage in which there was an Orofacial Stimulation Test System. The Orofacial Stimulation Test System had a drinking window for the rat head to enter and acquire a reward (30% sweetened condensed milk, Nestle Carnation^®^). The milk was placed in a cylindrical plastic container with a metal nipple drinker being located inside the drinking window. The Orofacial Stimulation Test System also consisted of a thermal module with its temperature being set at 24°C for training sessions and at 17 or 12°C for cold stimulation. An infrared beam was built in the drinking window and wired to a computer to automatically detect the head accessing the nipple drinker. A training session was started by placing a rat in the cage. After the rat was given 10 min to familiarize itself with its environment, the drinking window was opened and the testing rat was subsequently timed for 10 min to allow drinking the milk.

After 2 weeks of the adaptation training, the rats were entered into two experimental groups, the neuropathic pain group induced by oxaliplatin (oxaliplatin group) and the trigeminal neuropathic pain group induced by infraorbital nerve chronic constrictive ligation (ION-CCI). For the oxaliplatin group [24], rats were injected with oxaliplatin intraperitoneally at a dose of 2 mg/kg (200 µl each rat) for five consecutive days (a total dose of 10 mg/kg). The controls for the oxaliplatin group were rats injected with the same amount of saline. For ION-CCI group, right infraorbital nerve of each rat was ligated to induce chronic constriction nerve injury as described previously [22, 23]. In brief, each rat was anesthetized with intraperitoneal injection of ketamine/xylazine cocktail (100 mg/kg:10 mg/kg). The skin above the right eye was shaved and the rat head was immobilized. A 2-cm curvilinear incision was made superior to the right orbital cavity. The infraorbital nerve, located on the floor of the maxillary bone, was freed from the surrounding connective tissues and two ligatures were made approximately 5 mm apart with a 5-0 absorbable chromic gut suture Superion^®^ [22]. The incision was closed with 6-0 non-absorbable braided silk suture. The controls were rats with sham surgery without any ligatures. During a 2-week healing period, the rats underwent 2–4 sessions of post-surgical adaption training performed in the same manner as the pre-surgical adaptation training.

For both oxaliplatin group and ION-CCI group, 1 day before the experiments the testing rats’ facial areas were shaved. Subsequent experiments were performed for orofacial operant tests at the thermal module temperatures of 24, 17, or 12°C. The operant tests were performed again at these temperatures after injecting retigabine or vehicle. Retigabine was administered intraperitoneally at the doses of 0.19, 0.59, 1.67 and 15 mg/kg and orofacial operant tests were performed between 30 and 120 min after retigabine administration.

### Data analysis

Whole-cell recording data were analyzed using Clampfit 9 software. For orofacial operant tests, the events of head assessing nipple drinker were detected by the infrared beam, recorded by a computer, and analysed by the Oro Software (Ugo Basile, Comerio VA, Italy). This computer software recorded and analysed several variables of the rat’s behaviour including the total time the beam was broken, also defined as the total contact time, and the total count, which can also be described as total contact number. Unless otherwise indicated, total contact time in a 10-min experimental session was used as orofacial operant behavioural parameters. Data were presented as Mean ± SEM, analysed by the paired or unpaired Student’s t test, *P < 0.05, **P < 0.01, and ***P < 0.001.

## Results

**Fig. 1:**
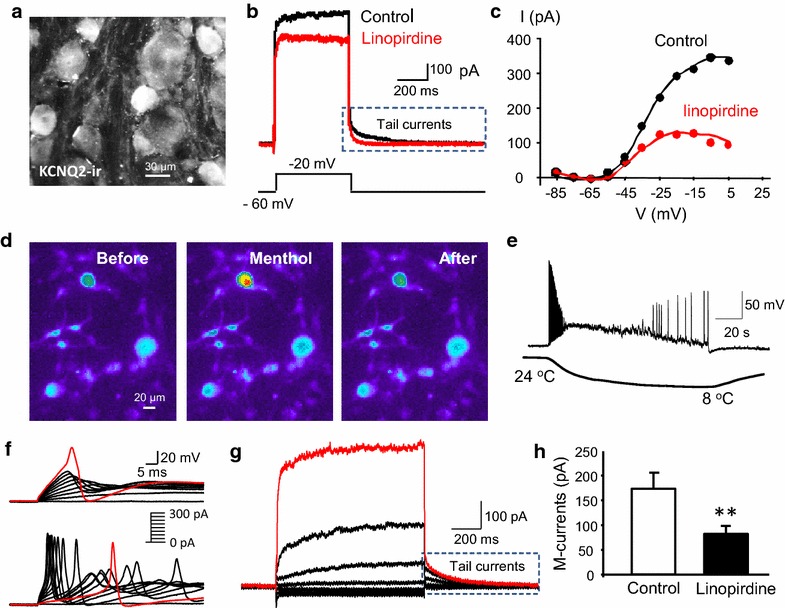
KCNQ channels in nociceptive cold-sensing trigeminal neurons. **a** Image shows KCNQ2 immunoreactivity (KCNQ2-ir) in a trigeminal ganglion section. **b** Sample traces of M-currents in the absence (*black*) and presence (*red*) of 20 µM linopirdine. The voltage step used to reveal M-currents was shown under the recording traces. The *box* indicates the M-currents, measured by the tail currents after the voltage step. **c** I–V curve of tail currents in the absence (*black*) and presence (*red*) of 20 µM linopirdine. **d** Ca^2+^-imaging shows an example of pre-identification of cold-sensing TG neurons with menthol (100 µM). **e** An example of a pre-identified cold-sensing TG neuron that responded to a cooling temperature ramp by firing action potentials. The cell was under the whole-cell current-clamp recording mode. The cooling ramp from 24 to 8°C is indicated under the recording trace. **f** Traces on the *top panel* show a nociceptive cold-sensing neuron that responds to 10 current-steps at the increment of 30 pA and the cell fires a slow action potential (Rheobase = 300 pA) with broad width and a hump in the repolarization phase. A total of 15 cold-sensing TG neurons belong to this category. Traces on the *bottom panel* show a non-nociceptive cold-sensing neuron that responds to 10 current-steps at the increment of 30 pA and the cell fires a fast action potential (Rheobase = 30 pA) without any hump in the repolarization phase. A total of 21 cold-sensing TG neurons belong to this category. **g** Traces show a nociceptive cold-sensing neuron that responds to voltage steps from −80 to −20 mV at 10 mV increment. The cell was held at −60 mV. **h** Summary result of M-currents recorded from nociceptive cold-sensing neurons in the absence (control) and presence of 20 µM linopirdine. The M-currents were measured by the deactivating tail currents following the voltage step of −20 mV. Data represent Mean ± SEM, **P < 0.01.

In trigeminal ganglion sections, strong immunoreactivity of KCNQ2 channels (KCNQ2-ir) could be observed in some but not all small-sized TG neurons (Fig. 1a). Consistently, patch-clamp recordings made from dissociated TG neurons showed the presence of M-type voltage-gated K^+^ currents (M-currents) in some small-sized TG neurons as demonstrated in Fig. 1b, c. The M-currents in TG neurons, as being revealed by the slow deactivating tail currents, could be largely inhibited by 20 µM linopirdine, a specific blocker of KCNQ channels (Fig. 1b, c). Previous studies have shown that only a small percentage of TG neurons are TRPM8-expressing cold-sensing sensory cells [5]. To determine whether M-currents were expressed in cold-sensing TG neurons, we used Ca^2+^-imaging technique to first identify cold-sensing TG neurons by testing their sensitivity to menthol, an agonist that activates the cold transducer TRPM8 channels (Fig. 1d). Similar to previous studies performed on DRG neurons by us and others [9, 11], almost all menthol-sensitive TG neurons were small-sized cells and these cells responded to 100 µM menthol by increasing intracellular Ca^2+^ levels (Fig. 1d). When patch-clamp recordings were applied to these menthol-sensitive TG neurons and recordings were performed under the whole-cell current-clamp mode, these TG neurons could be depolarized and fire action potentials following the application of a cold temperature ramp from 24 to 8°C (Fig. 1e). Of 36 menthol-sensitive TG neurons pre-identified by the Ca^2+^-imaging technique, patch-clamp recordings showed that 15 cells fired slow action potentials. The slow action potential had broad action potential width and a hump in repolarization phase (Fig. 1f, top panel; Table 1). These cells were previously characterized as nociceptive cold-sensing neurons [9]. The remaining 21 cells showed fast action potentials without a hump in action potential repolarization phase (Fig. 1f, bottom panel; Table 1). These cells are non-nociceptive cold-sensing neurons based on previous studies [9, 10]. Of the 21 non-nociceptive cold-sensing TG neurons, 7 cells (33%) did not express detectable M-currents and the remaining 14 cells (66%) showed M-currents. Overall, the amplitude of M-currents, measured by tail currents of deactivation from −20 to −60 mV, was 46.6 ± 8.9 pA (n = 21) in non-nociceptive cold-sensing neurons. In contrast, all 15 nociceptive cold-sensing TG neurons showed significant amounts of M-currents (Fig. 1g, h). The amplitude of M-currents was 134.5 ± 24.5 pA (n = 15) in nociceptive cold-sensing neurons, significantly higher than M-current amplitude of non-nociceptive cold-sensing TG neurons (P < 0.001, Table 1). Nociceptive cold-sensing TG neurons also had more negative resting membrane potentials and much higher rheobase in comparison with non-nociceptive cold-sensing TG neurons (Fig. 1f; Table 1). M-currents of nociceptive cold-sensing neurons were significantly inhibited by 20 µM linopirdine (Fig. 1h); the M-current amplitude was 173.6 ± 32.3 pA in the absence of linopirdine (n = 9) and 82.2 ± 16.7 pA in the present of linopirdine (n = 9, P < 0.01). Similarly, M-currents in non-nociceptive cold-sensing TG neurons were also significantly inhibited by 20 µM linopirdine (Table 1).

**Table 1:** M-currents, resting membrane potentials and rheobase of cold-sensing TG neurons

	AP type	M-current amplitude (pA)			RMP (mV)			Rheobase (pA)		
		All cells	Cntl	+Lino	All cells	Cntl	+Lino	All cells	Cntl	+Lino
NoCTG	Slow with hump	134 ± 24^###^, n = 15	174 ± 32, n = 9	82 ± 17**, n = 9	−66 ± 1^#^, n = 15	−67 ± 2, n = 8	−60 ± 2**, n = 8	426 ± 105^###^, n = 15	660 ± 164, n = 7	381 ± 96*, n = 7
NnCTG	Fast no hump	47 ± 9, n = 21	41 ± 10, n = 17	18 ± 6***, n = 17	−62 ± 1, n = 21	−62 ± 1, n = 17	−58 ± 2***, n = 17	92 ± 17, n = 21	101 ± 22, n = 17	87 ± 21^ns^, n = 17

Data represent Mean ± SEM.

*NoCTG* nociceptive cold-sensing trigeminal ganglion neurons, *NnCTG* non-nociceptive cold-sensing trigeminal ganglion neurons, *AP* action potential, *RMP* resting membrane potential, *Cntl* control without linopirdine, *+Lino* in the present of 20 µM linopirdine.

* P < 0.05, ** P < 0.01, *** P < 0.001, ns, no significant difference control vs +Lino, paired Student’s t test; ^#^ P < 0.05, ^###^ P < 0.01, NoCTG group vs NnCTG group, unpaired Student’s t test.

**Fig. 2:**
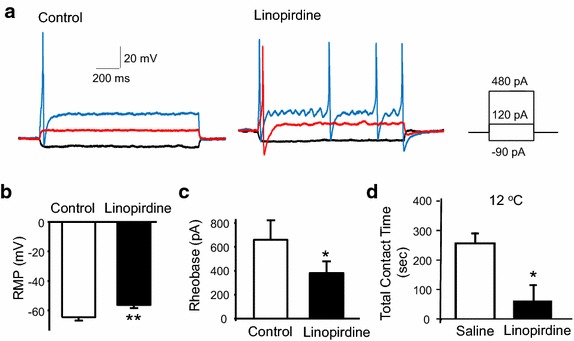
Increases of the excitability of nociceptive cold-sensing trigeminal neurons by inhibiting KCNQ channels. **a** An example shows that blocking KCNQ channels by linopirdine (20 µM) enhances excitability of nociceptive cold-sensing TG neurons. **b** Summary of the changes of resting membrane potentials (RMP) following the application of 20 µM linopirdine (n = 8). **c** Summary of the changes of rheobase for action potential firing following the application of 20 µM linopirdine (n = 7). **d** Behavioral cold hypersensitivity induced by linopirdine. Orofacial operant tests were performed at 12°C following the subcutaneous injection of saline or 0.39 mg linopirdine in oral facial regions. The linopirdine-injected animals (n = 4) show a significant reduction of total contact time in comparison with saline controls (n = 4). Data represent Mean ± SEM, *P < 0.05, **P < 0.01.

**Fig. 3:**
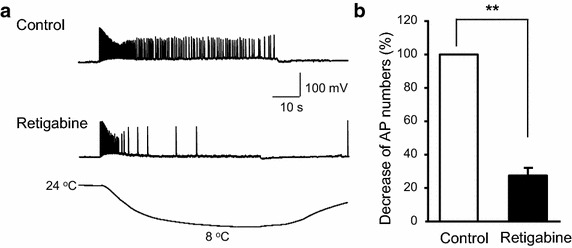
Suppression by retigabine of cold-induced action potential firing in nociceptive cold-sensing trigeminal neurons. **a** Sample traces show an example of cold-evoked action potential firing in a nociceptive cold-sensing TG neuron in the absence (control) and presence of 10 µM retigabine. Recordings were made under the whole-cell current-clamp mode. Cooling temperature ramp was applied from 24 to 8°C as indicated under the recording traces. **b** Summary data shows significant reduction of cold-evoked action potential firing in nociceptive cold-sensitive TG neurons (n = 8). Action potential numbers are normalized to control value. Data represent Mean ± SEM, **P < 0.01.

Inhibition of M-currents by linopirdine was associated with a significant reduction (less negative) of resting membrane potentials in nociceptive cold-sensing TG neurons (Fig. 2a, b); the resting membrane potentials were −66.9 ± 1.7 mV (n = 8) in the absence of and −60.2 ± 2.0 mV (n = 8, P < 0.01) in the presence of 20 µM linopirdine. This result suggests that KCNQ channels are involved in setting resting membrane potentials in nociceptive cold-sensing TG neurons. Inhibition of M-currents by linopirdine also increased the excitability of nociceptive cold-sensing TG neurons as was evidenced by the significant reduction of the rheobase for evoking action potential firing following the application of linopirdine (Fig. 2a, c); The rheobase was 660.0 ± 164.0 pA (n = 7) before and 381.4 ± 95.9 pA (n = 7, P < 0.05) following the application of 20 µM linopirdine. Nociceptive cold-sensing neurons were much less excitable than non-nociceptive cold-sensing neurons as was evidenced by more than four times higher rheobase (Table 1). In the non-nociceptive cold-sensing TG neurons, linopirdine also significantly decreased resting membrane potentials but rheobase was not significantly changed (Table 1). We next determined whether inhibition of KCNQ channels may result in behavioral cold hypersensitivity in orofacial regions. Linopirdine (0.39 mg, 100 µl) was subcutaneously injected into the orofacial regions of rats and orofacial operant tests were then performed 20 min later on these animals. As shown in Fig. 2d, when tested at 12°C, linopirdine injection led to a significant reduction of total contact time (265.0 ± 21.0 s with saline injection vs 62.0 ± 52.4 s with linopirdine injection, n = 4; P < 0.05). We then examined whether retigabine, a KCNQ2/3 channel potentiator, may suppress cold-induced action potential firing in nociceptive cold-sensing TG neurons. In this set of experiments, pre-identified nociceptive cold-sensing TG neurons were recorded under whole-cell current-clamp mode and action potential firing in these neurons was induced by the application of a cold ramp from 24 to 8°C. As shown in Fig. 3a, b, retigabine (10 µM) suppressed the number of cold-induced action potentials to 27 ± 4.6% of controls (n = 8, P < 0.01).

**Fig. 4:**
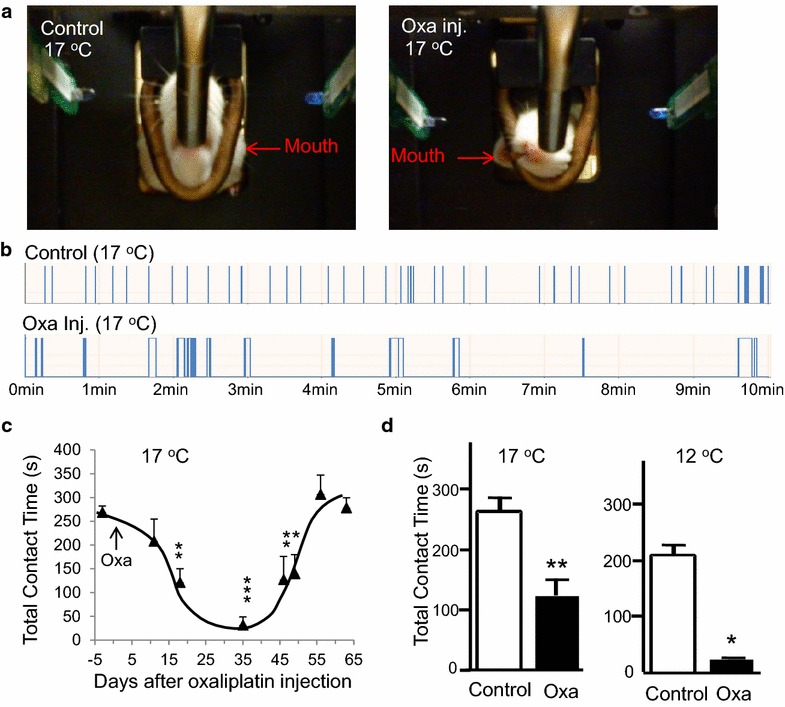
Orofacial operant assessment of oxaliplatin-induced orofacial cold allodynia/hyperalgesia. **a** Images show the postures commonly seen during orofacial operant tests at 17°C for control rats (*left*) and rats after oxaliplatin injections (*right*). The control rat rested its face on the cooling module while drinking milk. The oxaliplatin-injected (18 days after the injections) rat did no contact the cooling thermal module but tried to bite it off. **b** Recordings of contact numbers and duration of each contact in orofacial operant tests at 17°C for the control rat (*top*) and the oxaliplatin-injected rat (*bottom*). The duration of each orofacial operant test session was 10 min. **c** Change of total contact time over days after the injections of oxaliplatin (n = 5–8). **d** *Left* total contact time at 17°C for the rats 18 day after injection of saline (*open bar*) or oxaliplatin (oxa, *closed bar*). *Right* similar to *left panel* except the orofacial operant tests were conducted at 12°C. Data represent Mean ± SEM, *P < 0.05, **P < 0.01, ***P < 0.001.

Since retigabine suppressed cold-evoked action potential firing in nociceptive cold-sensing TG neurons, we sought to further explore whether the KCNQ2/3 channel potentiator could be of therapeutic use to alleviate trigeminal neuropathic pain that manifests as orofacial cold allodynia/hyperalgesia. We utilized two neuropathic pain animal models to test this idea. One was the ION-CCI model, which has been shown to induce cold allodynia/hyperalgesia as demonstrated by using orofacial operant tests in our previous studies [22, 23]. The other one was oxaliplatin-induced neuropathic pain model (oxaliplatin model), which had not been previously characterized using orofacial operant tests. In our oxaliplatin model, orofacial operant tests revealed cold allodynia/hyperalgesia in these animals several days after five consecutive injections of oxaliplatin (Fig. 4). Cold allodynia/hyperalgesia was evidenced by the presence of abnormal operant behaviors in these oxaliplatin-injected animals, e.g. trying to bite off cold thermal module in order to drink milk without getting cold stimulation (Fig. 4a). Control animals on the other hand had never behaved in such a manner. There was a significant reduction of total contact time during the orofacial operant tests performed at cooling temperatures of 17 and 12°C (Fig. 4b, d). Significant reduction of total contact time was observed 18 days after the injections of oxaliplatin, and the effect lasted for about 4 weeks (Fig. 4c). Figure 4d shows the comparison between control and oxaliplatin-injected animals for their total contact time during the orofacial operant tests. At cooling temperatures of 17°C the total contact time was 121.4 ± 28.6 s (n = 6) in oxaliplatin group, significantly shorter than the control group injected with saline (259.3 ± 27.1 s, n = 5, P < 0.01). More substantial difference in total contact time between oxaliplatin group (37.5 ± 14.9 s, n = 8) and control group (212.5 ± 15.0 s, n = 8, P < 0.05) was observed at the cooling temperature of 12°C. The very short contact time at 12°C in oxaliplatin-injected animals indicated that 12°C was too noxiously cold to be tolerated by these animals.

**Fig. 5:**
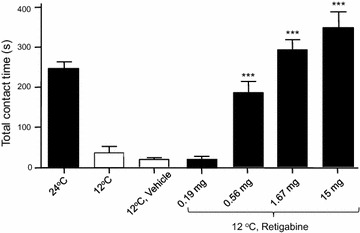
Alleviation of oxaliplatin-induced orofacial cold hyperalgesia by retigabine. *Bar graph* shows total contact time of orofacial operant tests for oxaliplatin-injected rats under the following conditions: 24, 12, 12°C with vehicle injection, 12°C with retigabine treatment at the dose of 0.19, 0.56, 1.67, and 15 mg/kg. Retigabine was administered intraperitoneally to the animals. Data represent Mean ± SEM, ***P < 0.01.

We used the oxilaplatin model and orofacial operant test to determine whether cold allodynia/hyperalgesia in these animals could be alleviated by retigabine. As shown in Fig. 5, the total contact time at the end of 10-min orofacial operant test session was 246.4 ± 17.8 s at 24°C and 37.5 ± 14.9 s at 12°C in rats 3 weeks after oxaliplantin injection (n = 8). Administration of vehicle did not significantly affect the total contact time at 12°C in these oxaliplatin-injected animals. In contrast, the total contact time at 12°C after the administration of retigabine was dose-dependently increased to 187.6 ± 26.4 s at a dose of 0.56 mg/kg (n = 8, P < 0.001), 294.5 ± 24.6 s at a dose of 1.67 mg/kg (n = 8, P < 0.001), and 350 ± 37.5 s at a dose of 15 mg/kg (n = 5, P < 0.001). At a low dose of 0.19 mg/kg, the total contact time at 12°C was 23 ± 5 s (n = 8), not significantly different from the total contact time at 12°C after vehicle injection.

**Fig. 6:**
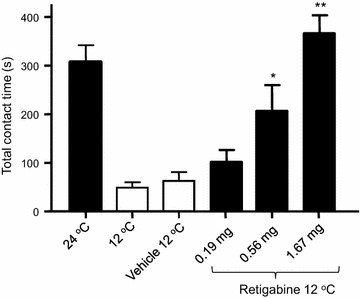
Alleviation by retigabine of orofacial cold hyperalgesia in infraorbital nerve chronic constrictive injury model. *Bar graph* shows total contact time of orofacial operant tests for infraorbital nerve chronic constrictive injury (ION-CCI) animals under the following conditions: 24, 12, 12°C with vehicle injection, 12°C with retigabine treatment at the doses of 0.19, 0.56, and 1.67 mg/kg. Retigabine was administrated intraperitoneal to the animals. Data represent Mean ± SEM, *P < 0.05; **P < 0.01.

For ION-CCI group, during post-operative period of 2–7 weeks, ION-CCI rats consistently showed cold allodynia/hyperalgesia in the orofacial operant tests as reported in our previous studies [22, 23]. Similar to our previous studies, ION-CCI rats displayed cold allodynial/hyperalgesia as shown in Fig. 4. At 24°C the total contact time at the end of 10-min orofacial operant test session was 307.2 ± 34.8 (n = 4) for postsurgery baseline. However, at 12 ◦C the total contact time was significantly reduced to 48.9 ± 11.3 s (n = 4, P < 0.01) in these ION-CCI rats. We examined whether cold allodynia/hyperalgesia in ION-CCI rats could be alleviated by retigabine, similar to what was observed in the oxaliplatin model. While the total contact time at 12°C was not significantly changed following the administration of vehicle (62.9 ± 17.9 s, n = 4) or low dose of retigabine at 0.19 mg/kg (102.3 ± 24.2 s, n = 4), the total contact time at 12°C was significantly increased to 206.9 ± 52.9 s (n = 4, P < 0.05) at a dose of 0.56 mg/kg, and to 366.5 ± 37 s (n = 4, P < 0.01) at a dose of 1.67 mg/kg (Fig. 6).

## Discussion

In the present study, we show the presence of functional KCNQ channels (M-currents) in nociceptive cold-sensing TG neurons and demonstrate their roles in setting the excitability and sensitivity of these sensory neurons to both electric and cooling stimuli. We further show in our in vivo behavioral experiments using orofacial operant tests that inhibition of KCNQ channels directly results in behavioral cold hypersensitivity. Furthermore, we demonstrate that potentiation of KCNQ channels alleviates cold allodynia/hyperalgesia in both oxaliplatin model and ION-CCI model of neuropathic pain. Our findings suggest that KCNQ channels may serve as an effective therapeutic target for the treatment of trigeminal neuropathic pain that manifests as orofacial cold allodynia/hyperalgesia.

KCNQ channel appears to be an essential molecule for nociceptive cold-sensing TG neurons since all nociceptive cold-sensing TG neurons showed M-currents in our study. In contrast, many non-nociceptive cold-sensing neurons did not display detectable M-currents. Furthermore, M-currents had higher amplitudes in nociceptive cold-sensing TG neurons than in non-nociceptive cold-sensing TG neurons. The high M-current amplitudes, i.e. high KCNQ channel expression, may help maintain the low excitability of nociceptive cold-sensing TG neurons to prevent innocuous cold from exciting and mild noxious cold from over-exciting these nociceptive neurons under physiological conditions. The expression of KCNQ channels was found to be highly regulated and previous studies showed that KCNQ channels in nociceptive DRG neurons were down-regulated under neuropathic and inflammatory conditions, which resulted in the hyperexcitability of nociceptive DRG neurons [14, 15, 17]. There is a possibility that KCNQ channels are also down-regulated in nociceptive cold-sensing TG neurons under pathological conditions, such as trigeminal neuropathy induced by chemotherapy and chronic trigeminal nerve injury and degeneration. A down-regulation of KCNQ channels in nociceptive cold-sensing TG neurons would increase the excitability of these cold-sensing neurons, which would lead to trigeminal neuropathic pain that manifests as cold allodynia/hyperalgesia. Consistent with this idea, we demonstrated that orofacial cold allodynia/hyperalgesia could be induced pharmacologically by inhibition of KCNQ channels with linopirdine. Furthermore, the KCNQ channel potentiator, retigabine, alleviated orofacial cold allodynia/hypealgeisa in the two neuropathic pain models used in the present study. However, the above putative mechanism will need to be confirmed by direct evidence showing KCNQ down-regulation at both protein and functional levels in these two animal models. There is another possibility that down-regulation of KCNQ did not occur in nociceptive cold-sensing TG neurons of our two animal models and orofacial cold allodynia/hyperalgesia was mediated by other mechanisms. In fact, upregulation of TRPM8 or TRPA1 has been previously suggested to be underlying mechanisms of cold allodynia/hyperalgesia after nerve injury and tissue inflammation [25, 26]. In addition, a recent study suggested that down-regulation of TREK1 and TRAAK potassium channels and up-regulation of HCN channels contributed to oxaliplatin-induced cold hypersensitivity [27]. Since KCNQ channel activation is downstream to cold transduction and membrane excitation, KCNQ channels should still be desirable therapeutic targets for effectively treating allodynia/hyperalgesia due to other mechanisms that cause cold hypersensitivity. Functional KCNQ channels in cold-sensing TG neurons may be KCNQ2/3 channels since KCNQ2-ir was detected in small sized TG neurons and KCNQ2/3 channels were found to be major KCNQ channels in nociceptive DRG neurons. Therefore, in addition to retigabine, other KCNQ2/3 channel potentiators may also be useful in treating orofacial cold allodynia/hyperalgesia.

We used ION-CCI model in the present study because it well represents chronic trigeminal nerve injury seen in human patients [22, 23]. We also used oxaliplatin model because oxaliplatin is the first line chemotherapy for advanced colorectal cancer, but orofacial cold allodynia and hyperalgesia are its major side-effects [28]. Orofacial operant tests were used in this study to allow us to measure pain rather than nociceptive reflex [22, 29]. This is an important advantage over classical behavioral tests, and our orofacial operant tests provide clinically relevant measures for the therapeutic effects of retigabine. The therapeutic effect of retigabin demonstrated in both models and with orofacial operant tests carries a great hope for patients with trigeminal neuropathic pain due to different causes. Retigabine has been approved by FDA for treating epilepsy clinically but it has side effects including dizziness and tremor due to its actions on KCNQ channels in the CNS. Future development of KCNQ potentiators that selectively act on KCNQ channels in the peripheral nervous system would help to limit their side effects.
